# The Association between Disseminated Intravascular Coagulation Profiles and Neurologic Outcome in Patients with In-Hospital Cardiac Arrest

**DOI:** 10.31083/j.rcm2509340

**Published:** 2024-09-23

**Authors:** Dong Hun Lee, Byung Kook Lee, Seok Jin Ryu, Ji Ho Lee, Sung Jin Bae, Yun Hyung Choi

**Affiliations:** ^1^Department of Emergency Medicine, Chonnam National University Hospital, 61469 Gwangju, Republic of Korea; ^2^Department of Emergency Medicine, Chonnam National University Medical School, 61469 Gwangju, Republic of Korea; ^3^Department of Emergency Medicine, Chung-Ang University Gwangmyeong Hospital, 14353 Gyeonggi-do, Republic of Korea

**Keywords:** in-hospital cardiac arrest, targeted temperature management, disseminated intravascular coagulation, scoring, prognosis

## Abstract

**Background:**

The relationship between disseminated intravascular coagulation (DIC) profiles and survival or neurological outcomes in out-of-hospital cardiac arrest (OHCA) patients is well known. In contrast, the relationship between DIC profiles and neurological outcomes in patients with in-hospital cardiac arrest (IHCA) remains unclear. This study sought to examine the correlation between DIC profiles and neurological outcomes in IHCA patients.

**Methods:**

A retrospective observational study was conducted on comatose adult IHCA patients treated with targeted temperature management between January 2017 and December 2022. DIC profiles were used to calculate the DIC score, and were measured immediately after the return of spontaneous circulation (ROSC). The primary endpoint was a poor neurological outcome at six months, defined by cerebral performance in categories 3, 4, or 5. Multivariate analysis was used to evaluate the association between DIC profiles and poor neurological outcomes.

**Results:**

The study included 136 patients, of which 107 (78.7%) patients demonstrated poor neurological outcomes. These patients had higher fibrinogen (3.2 g/L vs. 2.3 g/L) and fibrin degradation product levels (50.7 mg/L vs. 30.1 mg/L) and lower anti-thrombin III (ATIII) levels (65.7% vs. 82.3%). The DIC score did not differ between the good and poor outcome groups. In multivariable analysis, fibrinogen (odds ratio [OR], 1.009; 95% confidence intervals [CI], 1.003–1.016) and ATIII levels (OR, 0.965; 95% CI, 0.942–0.989) were independently associated with poor neurological outcomes.

**Conclusions:**

Decreased fibrinogen and ATIII levels after ROSC were an independent risk factor for unfavorable neurological outcomes in IHCA. The DIC score is unlikely to play a significant role in IHCA prognosis in contrast to OHCA.

## 1. Introduction

In-hospital cardiac arrest (IHCA) occurs in approximately 9–10 out of 1000 hospitalized patients [1]. Outcome-related factors of IHCA patients can help develop critical care plans for both patients and their families. These factors can help narrow the range of medical resources required for treatment [2]. Therefore, research analyzing factors related to neurological outcomes in IHCA patients could improve their prognosis through the appropriate distribution of medical resources.

There are differences in management between IHCA and out-of-hospital cardiac arrest (OHCA) patients. Survival rates and neurological outcomes in OHCA patients are significantly influenced by factors directly related to cardiac arrest management, such as high-quality cardiopulmonary resuscitation (CPR), the presence of a witness, appropriate application of defibrillation, and professional airway management [3, 4]. In contrast, in IHCA patients, most outcomes can be determined by the preexisting illness or the reason for hospitalization rather than the etiology of the cardiac arrest [5]. Therefore, pathological changes such as underlying conditions or alterations in laboratory tests during hospitalization might play a more crucial role in predicting future outcomes for IHCA patients.

When cardiac arrest patients experience the return of spontaneous circulation (ROSC), the resulting systemic ischemia-reperfusion response can induce increased systemic inflammation and coagulation, leading to disseminated intravascular coagulation (DIC) [6]. In addition to tissue hypoxia, this causes coagulation and immune dysfunction, potentially resulting in multiple organ failure and increased inflammation. In OHCA patients, altered DIC profiles and elevated DIC scores were associated with mortality or poor neurological outcomes [7, 8]. In contrast, the relationship between DIC profiles and neurological outcomes in IHCA patients remains unclear. Therefore, we sought to examine the correlation between DIC profiles and neurological outcomes in IHCA patients.

## 2. Materials and Methods

### 2.1 Study Design and Population

This retrospective observational study included comatose adult (age ≥18 years) IHCA patients who were treated with targeted temperature management (TTM) at the Chonnam National University Hospital, Gwangju, Korea, from January 2017 to December 2022. TTM was applied to all IHCA patients using the Arctic Sun® feedback-controlled surface cooling device (Arctic Sun™ 5000, Medivance Incorporated, Louisville, CO, USA). Patients with insufficient blood sampling and those with incomplete data sets were excluded. The Institutional Review Board (IRB) of the Chonnam National University Hospital Biomedical Research Institute approved this study (CNUH-2022-231). Due to the study’s retrospective nature, informed consent was not obtained.

### 2.2 Data Collection

We retrieved data from hospital records, collecting parameters such as age, sex, body mass index, preexisting medical conditions, witnessed collapse, initial monitored rhythm, diagnosis upon hospital admission, etiology of cardiac arrest, and the interval from collapse to ROSC. A Good Outcome Following Attempted Resuscitation (GO-FAR) was derived from the patients’ age and pre-arrest clinical characteristics [9]. The reasons for hospitalization before cardiac arrest, major trauma, stroke, and septicemia were also reviewed.

All laboratory findings were obtained immediately after ROSC. Post-ROSC laboratory findings were also recorded, including serum lactate and glucose levels, partial oxygen pressure (PaO_2_), partial carbon dioxide pressure (PaCO_2_), white blood cell count, hemoglobin, platelet count, activated partial thromboplastin time (APTT), international normalized ratio of prothrombin time (PT-INR), fibrinogen level, fibrin degradation product (FDP) level, D-dimer level, anti-thrombin III (ATIII) level, and target temperature of TTM. The DIC score was calculated using the methods suggested by the International Society of Thrombosis and Hemostasis (ISTH) [10]. Overt DIC was defined by a DIC score ≥5 [10]. The use of extracorporeal cardiopulmonary resuscitation (ECPR) during arrest and continuous renal replacement therapy (CRRT) were also recorded. We collected cumulative vasopressor indexes (CVIs) within 24 h of ROSC [11].

Neurological outcomes post-cardiac arrest were evaluated at six months via telephonic interviews, using the cerebral performance category (CPC) scale (CPC 1: good performance, CPC 2: moderate disability, CPC 3: severe disability, CPC 4: vegetative state, or CPC 5: brain death or death) [12]. The primary outcome was defined as a poor neurological status, indicated by a CPC of 3–5.

### 2.3 Statistical Analysis

We denoted categorical variables as frequencies and percentages, while continuous variables not conforming to the normality test were displayed as median values and interquartile ranges. We conducted χ^2^ tests with continuity correction within 2 × 2 tables to compare categorical variables across groups. Mann-Whitney U tests were employed for the comparison of continuous variables between groups.

Multivariate logistic regression analysis was applied to determine the association between DIC profiles and adverse neurological outcomes. Multicollinearity between variables was assessed before modeling. Variables yielding a *p* < 0.10 in the univariate comparisons were incorporated into the multivariate regression model. Using a backward stepwise methodology, variables were progressively discarded with a set threshold of *p* > 0.10 to devise a refined regression model. Outcomes of the logistic regression analysis were delineated as odds ratios (ORs) and 95% confidence intervals (CIs). We evaluated the predictive accuracy of fibrinogen and ATIII levels pertaining to adverse neurologic outcomes by using the area under the receiver operating characteristic curve (AUC). All analyses were performed using PASW Statistics for Windows, version 18.0 (SPSS, Inc., Chicago, IL, USA) and MedCalc version 19.0 (MedCalc Software, bvba, Ostend, Belgium). The threshold for statistical significance was established at *p* < 0.05 (two-sided).

## 3. Results

### 3.1 Patient Characteristics

**Fig. 1.**
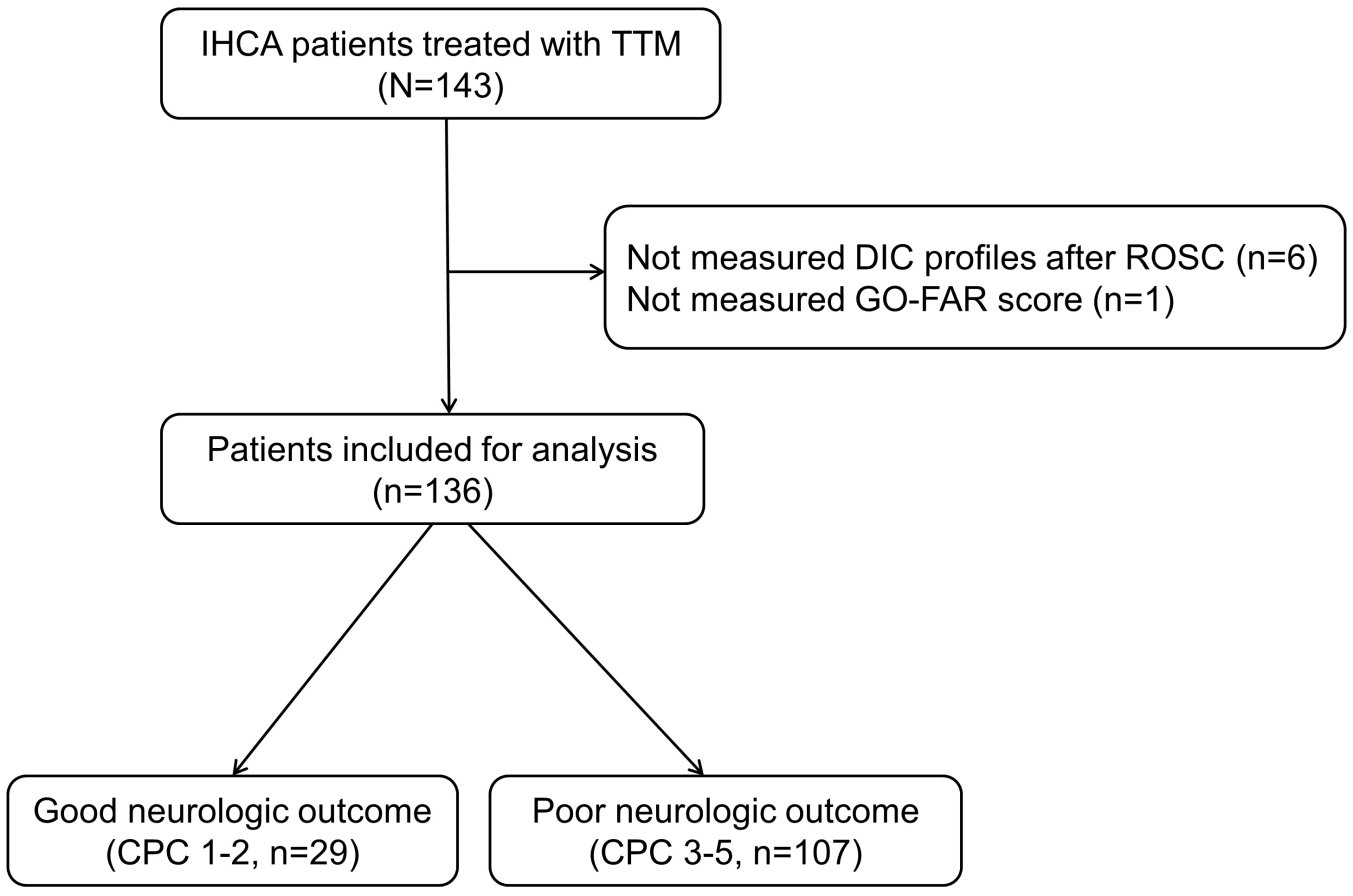
**Flow diagram of patient inclusion**. IHCA, in-hospital cardiac arrest; TTM, targeted temperature management; DIC, disseminated intravascular coagulation; ROSC, restoration of spontaneous circulation; GO-FAR score, Good Outcome Following Attempted Resuscitation score; CPC, cerebral performance category.

We identified 143 IHCA survivors who underwent TTM during the study period, and 136 patients satisfied the inclusion criteria (Fig. 1). Six patients were excluded due to failed blood sampling for ATIII, and one patient was excluded because of an unmeasurable GO-FAR score due to absent medical records at admission. The median age was 68.5 years, and 80 patients (58.8%) were male. Moreover, 114 patients (83.8%) had a witnessed cardiac arrest, and 33 patients (24.3%) had a shockable rhythm. In 51 patients (39.7%), IHCA resulted from a cardiac etiology. The median interval from collapse to ROSC was 14.5 minutes (7.0–26.3 minutes). At six months, 107 patients (78.7%) demonstrated poor outcomes (Table 1).

**Table 1.** **Comparisons of baseline characteristics according to neurological outcomes at 6 months**.

Variables		Total (N = 136)	Good (N = 29)	Poor (N = 107)	*p*
Demographics					
	Age, years	68.5 (58.0–76.8)	59.0 (52.0–70.5)	70.0 (60.0–79.0)	0.003
	Male, n (%)	80 (58.8)	14 (48.3)	66 (61.7)	0.276
Preexisting illness, n (%)					
	Coronary artery disease	17 (12.5)	2 (6.9)	15 (14.0)	0.476
	Congestive heart failure	24 (17.6)	3 (10.3)	21 (19.6)	0.374
	Hypertension	82 (60.3)	17 (58.6)	65 (60.7)	1.000
	Diabetes	52 (38.2)	10 (34.5)	42 (39.3)	0.800
	Chronic lung disease	20 (14.7)	5 (17.2)	15 (14.0)	0.889
	Renal impairment	24 (17.6)	3 (10.3)	21 (19.6)	0.374
	Liver cirrhosis	4 (2.9)	0 (0.0)	4 (3.7)	0.662
	Cerebrovascular accident	18 (13.2)	2 (6.9)	16 (15.0)	0.408
	Malignancy	13 (9.6)	5 (17.2)	8 (7.5)	0.219
Cardiac arrest characteristics					
	Witnessed collapse, n (%)	114 (83.8)	27 (93.1)	87 (81.3)	0.213
	Shockable rhythm, n (%)	33 (24.3)	11 (37.9)	22 (20.6)	0.091
	Presumed cardiac cause, n (%)	51 (37.5)	15 (51.7)	36 (33.6)	0.117
	Interval from collapse to ROSC, min	14.5 (7.0–26.3)	10.0 (5.0–15.0)	15.0 (10.0–30.0)	0.005
GO-FAR score		6 (−4–13)	−3 (−11–3)	9 (0–13)	<0.001
ECPR, n (%)		15 (11.0)	3 (10.3)	12 (11.2)	1.000
Target temperature of TTM					0.717
	33 °C, n (%)	102 (75.0)	23 (79.3)	79 (73.8)	
	36 °C, n (%)	34 (25.0)	6 (20.7)	28 (26.2)	
CRRT		50 (36.8)	6 (20.7)	44 (41.4)	0.071
CVI score		2 (2–4)	2 (2–4)	3 (2–5)	0.016

ROSC, return of spontaneous circulation; GO-FAR score, Good Outcome Following Attempted Resuscitation score; ECPR, extracorporeal cardiopulmonary resuscitation; CRRT, continuous renal replacement therapy; CVI, cumulative vasopressor index; TTM, targeted temperature management.

### 3.2 Comparative Analysis between Patients with Good and Poor Outcomes

Patients with poor outcomes were older, had a longer interval from collapse to ROSC, and elevated GO-FAR scores compared to patients with good outcomes (Table 1). There were no differences in ECPR, target temperature, and CRRT between patients with good outcomes and patients with poor outcomes. Patients with poor outcomes had higher CVI scores compared to patients with good outcomes. Immediately after ROSC, patients with poor outcomes had increased fibrinogen levels (3.2 g/L vs. 2.3 g/L) and FDP levels (50.7 mg/L vs. 30.1 mg/L) and decreased ATIII levels (65.7% vs. 82.3%) compared to patients with good outcomes (Table 2). There was no difference in DIC score and proportion of overt DIC between good and poor outcome groups.

**Table 2.** **Comparisons of laboratory parameters after ROSC according to neurological outcomes at discharge**.

Variable	Total (N = 136)	Good (n = 29)	Poor (n = 107)	*p*
Lactate, mmol/L	7.4 (4.0–12.7)	7.4 (4.6–11.8)	7.2 (3.9–13.4)	0.786
Glucose, mg/dL	207 (142–294)	236 (145–312)	204 (138–292)	0.483
PaO_2_, mmHg	179.3 (89.5–310.8)	274.0 (114.0–363.0)	148.0 (86.0–252.0)	0.082
PaCO_2_, mmHg	41.0 (29.0–58.0)	41.0 (27.5–53.1)	41.0 (30.0–58.0)	0.357
White blood cell count, × 10^9^/L	15.1 (10.7–23.2)	13.8 (11.3–22.2)	15.3 (10.5–23.3)	0.934
Hemoglobin, g/dL	11.0 (9.6–12.8)	11.7 (9.8–13.2)	10.9 (9.6–12.6)	0.318
Platelet count, × 10^9^/L	199 (135–290)	227 (164–312)	192 (127–284)	0.207
APTT, s	31.4 (28.1–41.6)	29.5 (26.3–43.3)	33.0 (28.3–41.6)	0.175
PT-INR	1.29 (1.15–1.61)	1.19 (1.07–1.53)	1.31 (1.19–1.62)	0.094
Fibrinogen, g/L	2.9 (2.1–4.0)	2.3 (1.8–3.3)	3.2 (2.3–4.2)	0.006
FDP, mg/L	44.9 (18.4–97.1)	30.1 (12.3–82.1)	50.7 (21.9–97.6)	0.037
D-dimer, mg/L	17.2 (6.9–35.2)	13.8 (4.2–27.6)	20.0 (7.6–35.2)	0.068
ATIII level, %	70.2 (55.4–82.0)	82.3 (70.4–93.4)	65.7 (51.9–78.1)	<0.001
DIC score	3 (3–3)	3 (3–3)	3 (3–3)	0.145
Overt DIC, %	6 (4.4)	1 (3.4)	5 (4.7)	1.000

ROSC, return of spontaneous circulation; PaO_2_, partial oxygen pressure; PaCO_2_, partial carbon dioxide pressure; APTT, activated partial thromboplastin time; PT-INR, international normalized ratio of prothrombin time; FDP, fibrin degradation product; DIC, disseminated intravascular coagulation; ATIII, anti-thrombin III.

### 3.3 Association between ATIII Level and Poor Neurological Outcome

In multivariable analysis, the interval from collapse to ROSC (OR, 1.080; 95% CI, 1.019–1.146), GO-FAR score (OR, 1.197; 95% CI, 1.100–1.302), fibrinogen level (OR, 1.012; 95% CI, 1.004–1.019), ATIII level (OR, 0.967; 95% CI, 0.942–0.994), and CVI score (OR, 1.886; 95% CI, 1.081–3.221) were independently associated with poor neurological outcomes in IHCA patients (Table 3). The AUCs of interval from collapse to ROSC, GO-FAR score, fibrinogen level, and ATIII level for poor neurological outcomes were 0.670 (95% CI, 0.585–0.749), 0.790 (95% CI, 0.712–0.855), 0.668 (95% CI, 0.582–0.746), and 0.736 (95% CI, 0.653–0.808) (Table 4).

**Table 3.** **Multivariate logistic regression analysis for poor neurological outcomes at 6 months**.

Variables	Unadjusted OR (95% CI)	*p*	Adjusted OR (95% CI)	*p*
Shockable rhythm	0.424 (0.175–1.026)	0.057	0.318 (0.072–1.414)	0.318
Interval from collapse to ROSC, min	1.050 (1.006–1.097)	0.025	1.080 (1.019–1.146)	0.010
GO-FAR score	1.071 (1.014–1.131)	0.014	1.197 (1.100–1.302)	<0.001
PaO_2_, mmHg	0.998 (0.995–1.000)	0.096	0.998 (0.993–1.003)	0.398
PT-INR	1.013 (0.894–1.148)	0.836	0.913 (0.806–1.034)	0.151
Fibrinogen, g/L	1.005 (1.001–1.009)	0.009	1.012 (1.004–1.019)	0.002
FDP, mg/L	1.003 (0.998–1.008)	0.280	1.004 (0.999–1.010)	0.107
D-dimer, mg/L	1.030 (0.997–1.065)	0.079	0.976 (0.911–1.046)	0.495
ATIII level, %	0.970 (0.950–0.990)	0.003	0.967 (0.942–0.994)	0.015
CRRT	2.677 (1.007–7.116)	0.048	2.511 (0.564–11.185)	0.227
CVI score	1.367 (1.063–1.758)	0.015	1.886 (1.081–3.221)	0.025

ROSC, return of spontaneous circulation; GO-FAR score, Good Outcome Following Attempted Resuscitation score; PaO_2_, partial oxygen pressure; PT-INR, international normalized ratio of prothrombin time; FDP, fibrin degradation product; ATIII, anti-thrombin III; CRRT, continuous renal replacement therapy; CVI, cumulative vasopressor index; OR, odds ratio; CI, confidence interval.

**Table 4.** **AUC analysis of interval from collapse to ROSC, GO-FAR score, fibrinogen level, and ATIII level for poor neurological outcomes at 6 months**.

Variable	AUC (95% CI)	*p*	Cut-off value	Sensitivity	Specificity
Interval from collapse to ROSC, min	0.670 (0.585–0.749)	0.003	>11	65.4	62.1
GO-FAR score	0.790 (0.712–0.855)	<0.001	>−1	75.7	72.4
Fibrinogen, g/L	0.668 (0.582–0.746)	0.002	>2.1	80.4	48.3
Anti-thrombin III, %	0.736 (0.653–0.808)	<0.001	≤73.2	68.2	72.4

AUC, area under the curve; ROSC, return of spontaneous circulation; GO-FAR score, Good Outcome Following Attempted Resuscitation score; ATIII, anti-thrombin III; CI, confidence interval.

### 3.4 Comparative Analysis of DIC Profiles and Poor Neurologic Outcome at 6 Months According to Major Trauma, Stroke, and Septicemia

**Supplementary Table 1** showed comparisons of coagulation parameters after ROSC and poor neurologic outcome at 6 months according to major trauma, stroke, and septicemia. ATIII levels of patients with stroke were higher than that of patients without stroke. Fibrinogen levels of patients with septicemia were higher than that of patients without septicemia. ATIII levels of patients with septicemia were lower than that of patients without septicemia. There was no difference in DIC score, overt DIC, and poor neurological outcome at 6 months according to major trauma, stroke, and septicemia.

## 4. Discussion

This study demonstrated that IHCA patients with poor neurological outcomes had lower levels of fibrinogen and ATIII compared to those with good neurological outcomes. Furthermore, fibrinogen and ATIII levels were strongly associated with poor neurological outcomes. ATIII level was weakly associated with poor neurological outcomes at 6 months, while fibrinogen level was not associated with poor neurological outcomes at 6 months.

ATIII, a single-chain protein found in plasma, is synthesized in the liver. It inhibits the coagulation system and is rapidly consumed in conditions such as systemic inflammatory response syndrome, sepsis, and DIC, leading to an early decrease in plasma levels [13]. In a study investigating the relationship between thrombin-antithrombin (TAT) levels and survival rates in patients resuscitated from cardiac arrest, the majority of patients exhibited elevated TAT levels [12]. Furthermore, increased TAT levels over the 24 hours following resuscitation were independently associated with survival [14]. Several studies assessed ATII levels in OHCA patients. In a study by Park *et al*. [15], ATIII levels did not differ between the good and poor outcome groups until 72 hours after ROSC [15]. In another study, when the normal cutoff value for ATIII was 20 mg/dL [16], the ATIII levels immediately after ROSC showed a normal range of over 75% in both good and poor outcome groups [7]. However, in the present study, the ATIII levels in the poor outcome group was 65.7%, which was lower than normal. Unlike in OHCA, the reason for hospitalization is closely related to the prognosis in IHCA. The reason for hospitalization in the poor outcome group might be more serious than in the good outcome group, which is reflected by the different GO-Far scores between the two groups. Low ATIII levels are associated with severity and mortality in critically ill patients [17, 18, 19]. Furthermore, ATIII activity might decrease with older age [20]. In the present study, the poor outcome group was older, and older age is a significant factor for prognosis following cardiac arrest. Therefore, the ATIII levels in the poor outcome group would be decreased before cardiac arrest compared to the good outcome group. Furthermore, the decrease in ATIII would be further accelerated due to the ischemic reperfusion injury from the cardiac arrest.

In OHCA patients, there was no difference in fibrinogen levels immediately after ROSC between good and poor outcome groups [21, 22]. In the present study, fibrinogen levels tended to be normal. In contrast to OHCA patients, the fibrinogen level in the poor outcome group was higher than that in the good outcome group, and this relationship continued following the multivariate analysis. Several studies [23, 24, 25] showed that mildly high fibrinogen levels are associated with the prognosis of critically ill patients. In patients with chronic heart failure, a high fibrinogen level (≥284 mg/dL) was associated with increased 90-day mortality [23]. Fibrinogen levels ≥350 mg/dL were associated with exacerbations of chronic obstructive pulmonary disease (COPD) and death in COPD patients [24]. Fibrinogen ≥280 mg/dL was associated with major adverse cardiovascular events, including death after percutaneous coronary intervention [25]. Fibrinogen plays a distinct role in arterial thrombosis through platelet aggregation, plasma viscosity, and fibrin formation, which increases cardiovascular risk [26, 27].

Unlike previous studies on OHCA, our study did not show a difference in DIC scores between good and poor outcomes groups of IHCA patients. The components of the ISTH score are platelet count, prothrombin time, fibrinogen, and D-dimer. In the present study, these elements did not differ between good and poor outcomes groups, except for fibrinogen levels. Fibrinogen exceeded 1.0 g/L in both groups; thus, no meaningful distinction could be made regarding the DIC score. In contrast to previous studies, D-dimer was significantly increased in both groups depending on the DIC score.

The present study has some limitations. First, this is a single-center retrospective study which is susceptible to bias. Therefore, future studies, including multi-center trials, are necessary to further validate our conclusions. Second, we did not directly compare patients who received TTM with those who did not, which could have affected the neurological outcomes. Previous study suggested that TTM might affect the prognosis of IHCA patients [28]. In the present study, we measured the DIC profile immediately after ROSC to minimize the interference of TTM, but future studies are needed to explore the effect of TTM on the DIC profile in both TTM and non-TTM groups of IHCA patients. Third, our study group consisted of only 29 patients. Thus, the ratio of the number of events per variable in the multivariate logistic regression model was lower than the desired ratio of >10 suggested by Peduzzi *et al*. [29]. The variables considered in the analysis were carefully selected. Additionally, the main purpose of this study was to examine the prognostic significance of DIC profiles, rather than to create a multivariable model to predict the outcome of IHCA patients. Therefore, the number of patients in the study group was limited. Fourth, there may be an interaction of major trauma, stroke, and septicemia for DIC profiles. However, all three diseases accounted for approximately 10% of the total patients and had no significant relationship with poor outcomes. Therefore, we believe that the three diseases had little effect on the relationship between prognosis and DIC profile in IHCA patients. Fifth, we did not investigate the relationship between the serial change of DIC profiles and the prognosis of IHCA patients. Finally, we did not consider blood transfusions or direct oral anticoagulants that could influence the DIC profile during post-cardiac arrest care. Therefore, the relationship with prognosis was investigated using only variables obtained after ROSC, and the DIC profile after ROSC was included in the regression model.

## 5. Conclusions

Decreased fibrinogen and ATIII levels after ROSC were independent risk factors for poor neurological outcomes in IHCA. Unlike OHCA, the DIC score is unlikely to play a significant prognostic role in IHCA. Future research is needed on the relationship between serial changes in the DIC profile after ROSC and neurological prognosis in IHCA patients.

## Availability of Data and Materials

All data generated or analyzed during this study are included in this article and its supplementary material files. Further enquiries can be directed to the corresponding author.

## Supplementary Material

Supplementary material associated with this article can be found, in the online version, at https://doi.org/10.31083/j.rcm2509340.
