# Supplementary material for: The Association between Disseminated Intravascular Coagulation Profiles and Neurologic Outcome in Patients with In-Hospital Cardiac Arrest
Source: Rev Cardiovasc Med. 2024 Sep 23;25(9):340. doi: 10.31083/j.rcm2509340 (PMC11440417; doi:10.31083/j.rcm2509340)
Supplement: Supplementary file 1 [file 2153-8174-25-9-340-s1.docx]

Supplementary Table 1. Comparisons of coagulation parameters after ROSC and poor neurologic outcome at 6 months according to major trauma, stroke, and septicemia.

|  | Major trauma | |  | Stroke | |  | Septicemia | |  |
| --- | --- | --- | --- | --- | --- | --- | --- | --- | --- |
| Variable | Yes (n = 10) | No (n = 126) | P | Yes (n = 7) | No (n = 129) | P | Yes (n = 19) | No (n = 117) | P |
| Platelet count, ×10^9^/L | 180 (147–230) | 200 (134–292) | 0.390 | 217 (169–303) | 197 (133–290) | 0.512 | 186 (124–315) | 200 (138–283) | 0.858 |
| APTT, s | 30.0 (24.3–36.6) | 32.0 (28.2–41.8) | 0.257 | 29.4 (28.2–36.5) | 31.4 (28.1–42.0) | 0.466 | 34.7 (31.3–43.5) | 30.5 (27.8–41.2) | 0.108 |
| PT-INR | 1.18 (1.03–1.47) | 1.30 (1.17–1.63) | 0.160 | 1.15 (1.04–1.28) | 1.30 (1.17–1.62) | 0.109 | 1.39 (1.22–1.90) | 1.27 (1.14–1.59) | 0.129 |
| Fibrinogen, g/L | 2.4 (1.6–2.7) | 3.0 (2.1–4.0) | 0.069 | 3.6 (2.6–5.2) | 2.9 (2.1–3.9) | 0.187 | 4.0 (2.8–4.8) | 2.9 (2.0–3.8) | 0.011 |
| FDP, mg/L | 30.8 (11.5–130.2) | 45.5 (18.9–97.7) | 0.617 | 49.8 (5.7–95.7) | 44.4 (18.7–97.7) | 0.890 | 62.2 (26.5–134.5) | 42.4 (16.5–85.1) | 0.053 |
| D-dimer, mg/L | 10.8 (4.0–29.8) | 18.8 (6.9–35.2) | 0.311 | 20.7 (1.6–35.2) | 17.1 (6.9–35.2) | 0.948 | 22.5 (9.4–35.2) | 16.4 (6.2–35.2) | 0.054 |
| Anti-thrombin III, % | 80.7 (48.3–90.4) | 69.2 (55.8–81.2) | 0.307 | 81.0 (72.3–94.7) | 68.9 (55.0–81.5) | 0.031 | 60.5 (46.4–69.2) | 72.0 (57.2–83.9) | 0.007 |
| DIC score | 3 (3–3) | 3 (3–3) | 0.391 | 3 (2–3) | 3 (3–3) | 0.080 | 3 (3–3) | 3 (3–3) | 0.673 |
| Overt DIC, % | 1 (10.0) | 5 (4.0) | 0.925 | 0 (0.0) | 6 (4.7) | 1.000 | 0 (0.0) | 6 (5.1) | 0.684 |
| Poor outcome, % | 9 (90.0) | 98 (77.8) | 0.612 | 7 (100.0) | 100 (77.5) | 0.347 | 18 (94.7) | 89 (76.1) | 0.123 |

ROSC: return of spontaneous circulation, PaO_2_: partial pressure of oxygen, PaCO_2_: partial pressure of carbon dioxide, aPTT, activated partial thromboplastin time, PT-INR: international normalized ratio of prothrombin time, FDP: fibrin degradation product, DIC: disseminated intravascular coagulation
